# A predictive model for osteoporosis in frail elderly patients with cerebral infarction: an integrated analysis based on machine learning and care-sensitive indicators

**DOI:** 10.3389/fendo.2026.1771964

**Published:** 2026-10-15

**Authors:** Yi Liu, Jing Wen, Yanxia Chen, Hui Li, Panpan Xie

**Affiliations:** 1 Department of Orthopedics, Zhenjiang Hospital Affiliated to Nanjing University of Chinese Medicine, Zhenjiang Hospital of Traditional Chinese Medicine, Zhenjiang, Jiangsu, China; 2 Department of Geriatrics, Zhenjiang Hospital Affiliated to Nanjing University of Chinese Medicine, Zhenjiang Hospital of Traditional Chinese Medicine, Zhenjiang, Jiangsu, China; 3 Department of Nursing, Zhenjiang Hospital Affiliated to Nanjing University of Chinese Medicine, Zhenjiang Hospital of Traditional Chinese Medicine, Zhenjiang, Jiangsu, China; 4 Department of Nephrology, Zhenjiang Hospital Affiliated to Nanjing University of Chinese Medicine, Zhenjiang Hospital of Traditional Chinese Medicine, Zhenjiang, Jiangsu, China

**Keywords:** cerebral infarction, elderly, frailty, osteoporosis, prediction model

## Abstract

**Objective:**

To identify key risk factors for osteoporosis in elderly patients with frailty and cerebral infarction using three machine learning algorithms—Least Absolute and Selective Operator (LASSO) regression, Extreme Gradient Boosting (XGBoost), and Random Forest (RF)—and establish a risk prediction model to provide data support for clinical prevention.

**Methods:**

A retrospective study was conducted using clinical data from 120 elderly frail patients with cerebral infarction admitted to our hospital between April 2024 and October 2025. Patients were categorized into an osteoporosis occurrence group (n=41) and a non-occurrence group (n=79) based on medical records indicating osteoporosis development. Clinical data were collected and compared. Risk factors were screened using “overlap-covering” screening with three machine learning methods: LASSO regression, XGBoost, and RF. The intersecting variables were incorporated into a logistic regression model to analyze risk factors for osteoporosis in elderly frail patients with cerebral infarction. Data analysis was performed using R software. RF to screen risk factors using “overlap coverage.” Intersecting variables were incorporated into logistic regression to analyze factors influencing osteoporosis in elderly patients with frailty and cerebral infarction. A nomogram prediction model was developed using R software, internally validated via Bootstrap resampling (1000 iterations), and its predictive accuracy assessed through calibration curves. Clinical utility was evaluated using decision curves.

**Results:**

Significant differences (P < 0.05) were observed between the occurrence and non-occurrence groups in gender, age, affected circulatory system, disease severity, Tilburg Frailty Inventory (TFI) score, Global Nutritional Risk Index (GNRI), Barthel Index, and Self-Rating Anxiety Scale (SAS) score. Through machine learning screening using LASSO regression, XGBoost, and RF, five common risk factors for osteoporosis in elderly frail patients with cerebral infarction were identified: age, TFI score, GNRI, Barthel Index, and SAS score as five common risk factors for osteoporosis in elderly frail patients with cerebral infarction. These were incorporated into a logistic regression model. Analysis revealed that age, TFI score, and SAS score were risk factors for osteoporosis in this population (OR = 1.102, 2.876, 1.177, P<0.05). GNRI and Barthel Index were protective factors (OR = 0.913, 0.838, P<0.05). The Hosmer-Lemeshow goodness-of-fit test yielded χ²=2.345, P = 0.969>0.05, indicating acceptable model fit. Based on the logistic regression results, a risk prediction model for osteoporosis in elderly frail patients with cerebral infarction was constructed using a nomogram. The AUC was 0.955 (95% CI: 0.920–0.991). Calibration curves showed Cox-Snell R² = 0.755 and Nagelkerke R² = 0.546, indicating strong explanatory power and high calibration of the model. The clinical decision curve generally exceeded both extreme curves, demonstrating that the nomogram effectively captured the risk distribution. Nagelkerke R²: 0.546, indicating strong explanatory power and high calibration for the dependent variable. The clinical decision curve generally exceeded both extreme curves, demonstrating that the factors included in the nomogram provide high net benefit for predicting osteoporosis in patients.

**Conclusion:**

Key factors influencing osteoporosis development in frail elderly patients with cerebral infarction include age, TFI score, GNRI, Barthel Index, and SAS score. The risk prediction model constructed using these factors demonstrates predictive value, high calibration accuracy, and clinical utility, providing theoretical guidance for clinical practice.

## 1 Introduction

As the global population continues to age at an accelerated pace, health issues among the elderly have emerged as a major public health challenge (1, 2). Wang J et al. (3) noted that against this backdrop, frailty in older adults—a clinical syndrome reflecting diminished physiological reserves and reduced stress resilience—has garnered significant attention due to its strong association with adverse health outcomes such as falls, hospitalizations, and mortality. Previous studies have indicated that cerebral infarction, as one of the common cerebrovascular diseases, is a primary cause of long-term disability and cognitive impairment in the elderly. It typically interacts with frailty: frail status significantly increases the risk of cerebral infarction and adverse outcomes, while cerebral infarction, accompanied by symptoms such as functional impairment and reduced activity, significantly accelerates the frailty process, thereby forming a complex vicious cycle (4).

Clinical studies indicate that among the vulnerable population of elderly patients with frailty and cerebral infarction, osteoporosis—a systemic skeletal disorder characterized by reduced bone mass, impaired bone microarchitecture, and increased bone fragility—may lead to prolonged bed rest, complications, and even a sharply elevated risk of mortality in the elderly (5). Therefore, accurately identifying key risk factors for osteoporosis in frail elderly patients with cerebral infarction is crucial for enabling early intervention and improving clinical outcomes. However, current clinical practice offers limited reports on risk assessment and management of osteoporosis in this population. Traditional risk assessment primarily relies on baseline data such as age and gender, yet fails to fully capture the nonlinear relationships among multidimensional indicators, resulting in limited predictive efficacy (6). In recent years, machine learning algorithms have demonstrated significant potential in constructing predictive models for medical applications. Compared to traditional statistical methods like logistic regression, machine learning algorithms better handle high-dimensional, nonlinear data with complex interactions, enabling more precise individualized risk prediction and offering new directions for developing specialized predictive tools (7, 8). In particular, ensemble machine learning models such as Random Forest and XGBoost have shown superior performance in clinical prediction by capturing complex variable interactions and improving model robustness (9, 10). Furthermore, translating the features identified by these algorithms into a visual nomogram not only enhances model interpretability but also provides clinicians with an intuitive tool for individualized risk assessment (11). Recent population-based studies have also demonstrated that machine learning approaches can outperform or complement traditional regression methods in predictive modeling, further supporting their integration into clinical research workflows (12). However, AltıntaşE et al. (13) argue that constructing efficient, scientifically sound predictive models requires not only precise machine algorithms but also comprehensive, predictive feature variables. Previous studies have predominantly focused on traditional clinical parameters like bone density, age, and gender, while care-sensitive indicators are often overlooked (14). Previous studies indicate that care-sensitive indicators dynamically reflect patients’ self-care capabilities, nutritional status, psychological state, and treatment adherence. They serve not only as sensitive gauges of overall health but may also hold potential associations with skeletal health (15). Therefore, systematically integrating care-sensitive indicators into predictive feature systems can effectively address the limitations of traditional clinical parameters, align with patients’ actual care and health management needs, and enhance the validity and clinical utility of predictive models.

Based on this, this study focuses on the specific population of elderly individuals with frailty and cerebral infarction to construct a precise, multidimensional osteoporosis risk prediction model. It innovatively integrates machine learning algorithms with multidimensional nursing-sensitive indicators, consolidating multi-dimensional data to identify high-risk groups and provide theoretical guidance for clinical practice.

## 2 Materials and methods

### 2.1 Ethical statement

This study was approved by the institutional review board and ethics committee. Given its retrospective nature and use of de-identified patient data, informed consent was not required as no risk or detriment to patients was anticipated. This exemption complies with regulations and ethical guidelines pertaining to retrospective research.

### 2.2 Study design

This retrospective study included clinical data from 120 elderly frail patients with cerebral infarction admitted to our hospital between April 2024 and October 2025. All patient data were extracted from the electronic medical record system. Patients were categorized into an osteoporosis occurrence group (n=41) and a non-occurrence group (n=79) based on medical record documentation of osteoporosis development.

### 2.3 Inclusion and exclusion criteria

Inclusion criteria: (1) Meeting criteria for cerebral infarction according to the Chinese Guidelines for Prevention and Treatment of Cerebrovascular Diseases (16), diagnosed via cranial CT or MRI imaging; (2) Meeting diagnostic criteria for geriatric frailty (Tilburg Frailty Inventory [TFI] score >5) (17); (3) Age ≥60 years; (4) Complete clinical documentation; (5) Stable vital signs.

Exclusion Criteria: (1) End-stage disease; (2) History of corticosteroid use or medications inducing osteoporosis; (3) Cognitive impairment; (4) History of stroke with residual neurological deficits affecting limb function; (5) Concurrent severe debilitating conditions such as infection or malignancy; (6) Recent fractures or concomitant cervical/lumbar spine disorders affecting mobility or peripheral nerves.

### 2.4 Basis for grouping

All subjects underwent dual-energy X-ray absorptiometry (enCORE, General Electric Company, version 15) to measure bone mineral density at the femoral neck, lumbar spine (L1-L4), and left total hip. Osteoporosis diagnosis criteria followed the definitions in the Chinese Geriatric Osteoporosis Diagnosis and Treatment Guidelines (2018) (18):T-score ≥ -1 indicates normal bone density; -2.5 < T-score < -1 indicated low bone mass; T-score ≤ -2.5 indicated osteoporosis.

### 2.5 General data collection

Patient demographics were collected from medical records, including gender (male/female), age, body mass index (BMI), smoking history (present if daily consumption > 1 cigarette pack-year and duration > 1 year or cessation < 1 year) (yes/no), alcohol consumption history (defined as daily intake > 1 drink unit for > 1 year or cessation < 1 year; 1 drink unit = 45mL spirits/360mL beer/120mL fruit wine) (yes/no), educational attainment (high school or below/college or above), disease duration, affected circulation system (multifocal infarction in unilateral anterior circulation/multifocal infarction in posterior circulation), disease severity (mild-moderate/severe), regular use of osteoporosis medication (yes/no), systolic blood pressure, diastolic blood pressure.

### 2.6 Laboratory indicators

Collect 5 mL of morning fasting venous blood from patients. Centrifuge at 3000 rpm for 10 minutes using a centrifuge (Model: LL900, Manufacturer: Luoyang Hongshi Machinery Equipment Co., Ltd.) to separate serum. Analyze serum using a fully automated biochemical analyzer (Model: Beckman AU5800, Country of Origin: USA) to measure triglycerides (TG), total cholesterol (TC), low-density lipoprotein cholesterol (LDL-C), high-density lipoprotein cholesterol (HDL-C), and fasting blood glucose (FBG).

### 2.7 TFI score

This scale, developed by foreign scholars including Gobbens, is a tool for assessing individual frailty. It comprises three dimensions—physical frailty, psychological frailty, and social frailty—totaling 15 items. Each item is scored 0–1 points, yielding a total score ranging from 0 to 15 points. Higher scores indicate more pronounced frailty in patients.

### 2.8 Nutritional status assessment

Assessment is conducted using the Global Nutritional Risk Index (GNRI) (19). GNRI is calculated based on parameters including the subject’s height (cm), weight (kg), ideal body weight (kg), and serum albumin (g/L). Calculation formula: GNRI = (1.489 × albumin (g/L)) + 41.7 × [weight/ideal weight]. Ideal weight (male) = Height (cm) - 100 - ([Height (cm) - 150]/4). When actual weight exceeds ideal weight, set Weight/Ideal weight = 1. GNRI nutritional risk categories: High risk (GNRI < 82), Moderate risk (82 ≤ GNRI < 92), Low risk (92 ≤ GNRI ≤ 98), No risk (GNRI > 98).

### 2.9 Activities of daily living assessment

Evaluated using the Barthel Index (20), comprising 10 items including toileting, bathing, dressing, and eating. Total score ranges from 0 to 100 points:100 points indicates complete independence;>60 points indicates basic self-care capability; 41–60 indicates moderate impairment requiring assistance; 20–40 indicates severe impairment requiring substantial assistance; <20 indicates complete disability requiring full dependence on others.

### 2.10 Anxiety assessment

The Self-Rating Anxiety Scale (SAS) (21) was used for assessment. This 20-item scale employs a 4-point rating system to evaluate the frequency of symptoms defined by the items. The criteria are as follows: 1 indicates “none or very little time,” 2 indicates “a small portion of the time,” 3 indicates “a considerable amount of time,” and 4 indicates “most or all of the time.” Scores are assigned according to this 1–4 sequence. The cutoff point for standardized scores is 50 points, with 50–59 points indicating mild anxiety, 60–69 points indicating moderate anxiety, and 70 points or above indicating severe anxiety.

### 2.11 Statistical analysis

Data statistical analysis and visualization were performed using SPSS 26.0 and R 4.3.2 software. For normally distributed quantitative data, independent samples t-tests were performed, with results presented as ( $\bar{x}$ ± s). Non-normally distributed data underwent Mann-Whitney U tests, displayed as [M (P25, P75)]. Categorical variables were compared using chi-square (χ²) tests, expressed as frequencies. The significance level was set at P < 0.05. Initial univariate screening was performed to identify variables with significant between-group differences, which served as the candidate pool for subsequent machine learning-based feature selection. We acknowledge that univariate pre-screening may occasionally exclude variables that are important through non-linear interactions or confounding effects; however, given the limited sample size, this step was deemed necessary to reduce the dimensionality of the feature space and maintain statistical efficiency in the downstream machine learning models. To mitigate the potential bias introduced by this pre-screening step, we conducted a sensitivity analysis in which all candidate variables (including those not significant in univariate analysis) were entered into the machine learning pipeline. The overlap-coverage results remained unchanged, and the final logistic regression model yielded consistent significant predictors, confirming the robustness of our findings. Nevertheless, we acknowledge that the pre-screening step remains a limitation, and future studies with larger sample sizes should consider omitting this step to allow for unbiased variable selection. Variable selection was then performed using the least absolute shrinkage and selection operator (LASSO) regression, extreme gradient boosting (XGBoost), and random forest (RF). For LASSO regression, the optimal regularization parameter lambda was determined via 10-fold cross-validation using the cv.glmnet function, with the lambda.min criterion selected to minimize the cross-validated binomial deviance. For XGBoost, hyperparameter tuning was performed using grid search with 10-fold cross-validation, exploring the following parameter space: nrounds (50, 100, 150, 200), max_depth (3, 4, 5, 6), eta (0.01, 0.05, 0.1, 0.3), gamma (0, 0.1, 0.2), colsample_bytree (0.6, 0.8, 1.0), min_child_weight (1, 2, 3), and subsample (0.6, 0.8, 1.0). The optimal hyperparameter combination was selected based on the highest cross-validated area under the receiver operating characteristic curve. For Random Forest, grid search with 10-fold cross-validation was similarly employed, tuning mtry (2, 3, 4, 5) and ntree (500, 1000, 1500), with the optimal model selected according to the highest cross-validated accuracy. Variable importance was quantified using the built-in feature importance metrics of each algorithm, and variables with a contribution greater than 5% were retained. The rationale for the ‘overlap-coverage’ screening strategy, which takes the intersection of variables identified by these three distinct algorithms, is to enhance the robustness and parsimony of the final model. LASSO is effective for linear feature selection through L1 regularization, while XGBoost and RF capture non-linear interactions and higher-order dependencies. By selecting only variables that are consistently identified as important across both linear and tree-based paradigms, we reduce the risk of algorithm-specific bias and overfitting, thereby selecting more reliable and generalizable predictors. To provide quantitative evidence for the stability of this strategy, we assessed the reproducibility of the finally selected variables by applying bootstrap resampling to the full dataset. Specifically, the feature selection procedure using LASSO, XGBoost, and RF with overlap-coverage was performed once on the full dataset to identify the final set of predictors. The stability of these selected variables was then evaluated by generating 1000 bootstrap resamples from the full dataset and determining, in each resample, whether each variable was identified as important by at least two of the three algorithms under the same pre-specified selection criteria. This stability assessment was not intended to re-run the complete feature selection pipeline within each bootstrap iteration; rather, it was used to quantify how consistently the finally selected variables would be prioritized across resampled datasets. The five variables ultimately selected (age, TFI score, GNRI, Barthel Index, and SAS score) were identified in 94.2%, 91.8%, 96.5%, 93.7%, and 92.4% of bootstrap iterations, respectively, by at least two of the three algorithms. In contrast, variables that were not included in the final model (gender, affected circulatory system, and disease severity) showed selection frequencies of 48.3%, 39.6%, and 42.1%, respectively. These results quantitatively demonstrate that the overlapping variables are consistently and robustly identified across resamples, supporting the stability and reliability of the overlap-coverage approach. This approach, while intentionally conservative, may miss predictors that are uniquely detected by a single algorithm; however, it prioritizes stability and interpretability over exhaustive feature inclusion. This aligns with best practices in clinical prediction modeling, where combining complementary algorithms can yield more stable feature sets, particularly in small- to-moderate sample settings. To ensure transparency and avoid overclaiming, we further clarify the following: the bootstrap resampling (1000 iterations) used for internal validation was performed solely for the final logistic regression model, specifically to estimate the optimism-corrected performance metrics (e.g., calibration slope and discrimination) using the rms::validate() function. This procedure does not re-run feature selection within each bootstrap iteration; rather, it re-fits the pre-specified model on each resampled dataset and evaluates its performance on the original dataset. We acknowledge that this approach may yield slightly optimistic performance estimates compared to a fully nested feature selection procedure. However, given the limited sample size and the stability of the selected variables (as demonstrated by the selection frequency analysis), we believe this trade-off is acceptable. Future studies with larger sample sizes should consider implementing a fully nested feature selection pipeline to further reduce potential bias. Selected variables were incorporated into logistic regression analysis. A prediction model based on the rms package was developed using scatter plots. Internal validation was conducted via bootstrap resampling (1000 iterations). Predictive accuracy was assessed using calibration curves, and clinical utility was evaluated through decision curves.

## 3 Results

### 3.1 Comparison of clinical data between the occurrence group and non-occurrence group of elderly patients with frailty and cerebral infarction

Comparing clinical data between the occurrence group and non-occurrence group of elderly patients with frailty and cerebral infarction, The results showed that there were no statistically significant differences between the occurrence group and the non-occurrence group in terms of BMI, smoking history, drinking history, and other clinically relevant data (P > 0.05). However, there were statistically significant differences between the occurrence group and the non-occurrence group in terms of gender, age, affected circulatory system, disease severity, TFI score, GNRI, Barthel Index, and SAS score (P < 0.05), as shown in **Table 1**.

**Table 1:** Comparison of relevant data of elderly frailty complicated with cerebral infarction in the occurrence group and the non-occurrence group.

Index		Occurrence group (n=41)	Non-occurrence group (n=79)	*χ*^2^/*t/Z*value	*P* value
Gender (n)	male	13	49	9.935	0.002
	female	28	30		
Age (years, * $\bar{x}$ ± s*)		79.46 ± 9.39	72.88 ± 8.14	3.982	<0.001
BMI (kg/m², * $\bar{x}$ ± s*)		25.16 ± 2.52	24.67 ± 2.74	0.954	0.342
History of smoking (n)	have	16	18	3.506	0.061
	no	25	61		
History of alcohol consumption (n)	have	12	15	1.636	0.201
	no	29	64		
Educational attainment (n)	High school and below	27	41	2.141	0.143
	College degree or above	14	38		
Course of the disease (years, * $\bar{x}$ ± s*)		2.53 ± 0.42	2.41 ± 0.40	1.532	0.128
The affected circulatory area (n)	Multiple infarctions in unilateral anterior circulation	24	28	5.862	0.015
	Multiple infarctions in the posterior circulation	17	51		
Severity of the disease (n)	Mild to moderate	22	61	7.023	0.008
	Severe	19	18		
Take anti-osteoporosis drugs regularly (n)	yes	25	60	2.929	0.087
	no	16	19		
Systolic blood pressure (mmHg, * $\bar{x}$ ± s*)		138.11 ± 15.34	138.06 ± 16.90	0.016	0.987
Diastolic blood pressure (mmHg, * $\bar{x}$ ± s*)		77.83 ± 5.96	79.63 ± 8.91	1.164	0.247
TG (mmol/L, * $\bar{x}$ ± s*)		1.56 ± 0.50	1.50 ± 0.44	0.676	0.500
TC (mmol/L, * $\bar{x}$ ± s*)		4.48 ± 1.02	4.52 ± 1.10	0.194	0.847
LDL-C (mmol/L, * $\bar{x}$ ± s*)		2.34 ± 0.35	2.26 ± 0.36	1.165	0.246
HDL-C (mmol/L, * $\bar{x}$ ± s*)		1.22 ± 0.28	1.28 ± 0.36	0.930	0.354
FBG (mmol/L, * $\bar{x}$ ± s*)		5.22 ± 1.08	5.11 ± 1.10	0.523	0.602
TFI score (points, *M (P25, P75)*)		9.00 (9.00, 10.00)	8.00 (7.00, 8.00)	5.376	<0.001
GNRI (* $\bar{x}$ ± s)*		76.05 ± 10.27	85.99 ± 9.35	5.339	<0.001
Barthel Index (points, * $\bar{x}$ ± s*)		38.33 ± 7.33	47.88 ± 8.85	6.055	<0.001
SAS score (points, * $\bar{x}$ ± s*)		66.78 ± 8.13	58.23 ± 8.77	5.190	<0.001

### 3.2 Screening key variables associated with osteoporosis in frail elderly patients with cerebral infarction using three machine learning algorithms

To optimize model performance and address multicollinearity, LASSO regression was employed for feature selection among preliminarily screened differential variables, compressing redundant regression coefficients to zero. To validate the reliability of the LASSO results, cvlassologit cross-validation was used to determine the optimal λ. Ultimately, the λ value yielding the smallest error in 10-fold cross-validation (λ=0.018) was selected as the optimal fitting parameter. Six variables—gender, age, TFI score, GNRI, Barthel Index, and SAS score (**Figure 1**). XGBoost ultimately incorporated seven important variables (contribution >5%), including gender, age, disease severity, TFI score, GNRI, Barthel Index, and SAS score (**Figure 2**). The RF model ultimately incorporated five important variables (contribution >5%): age, TFI score, GNRI, Barthel Index, and SAS score (**Figure 3**). An “overlap coverage” analysis of the important variables identified by the three machine learning models revealed five significant factors: age, TFI score, GNRI, Barthel Index, and SAS score.

**Figure 1:**
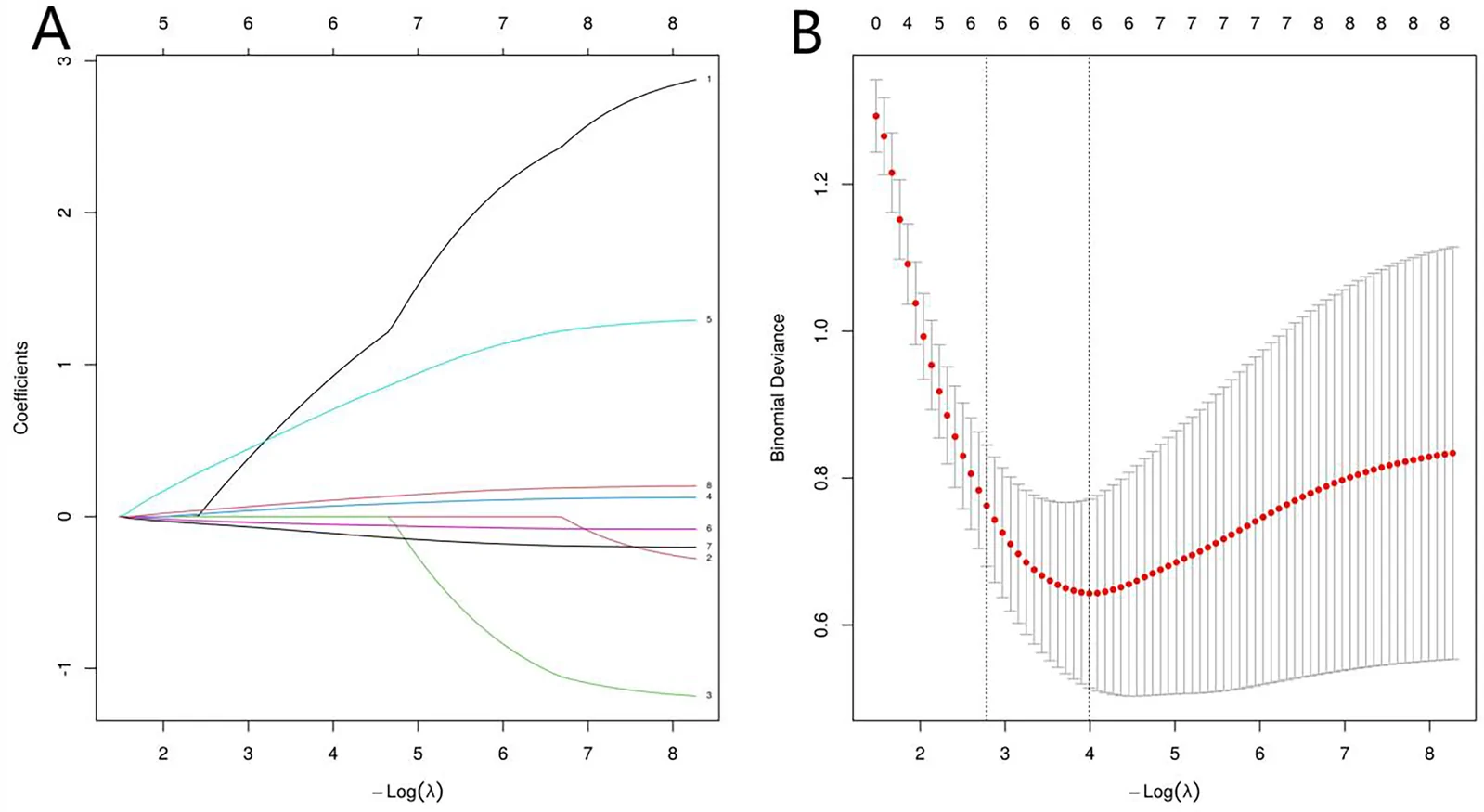
*LASSO* regression analysis chart.

**Figure 2:**
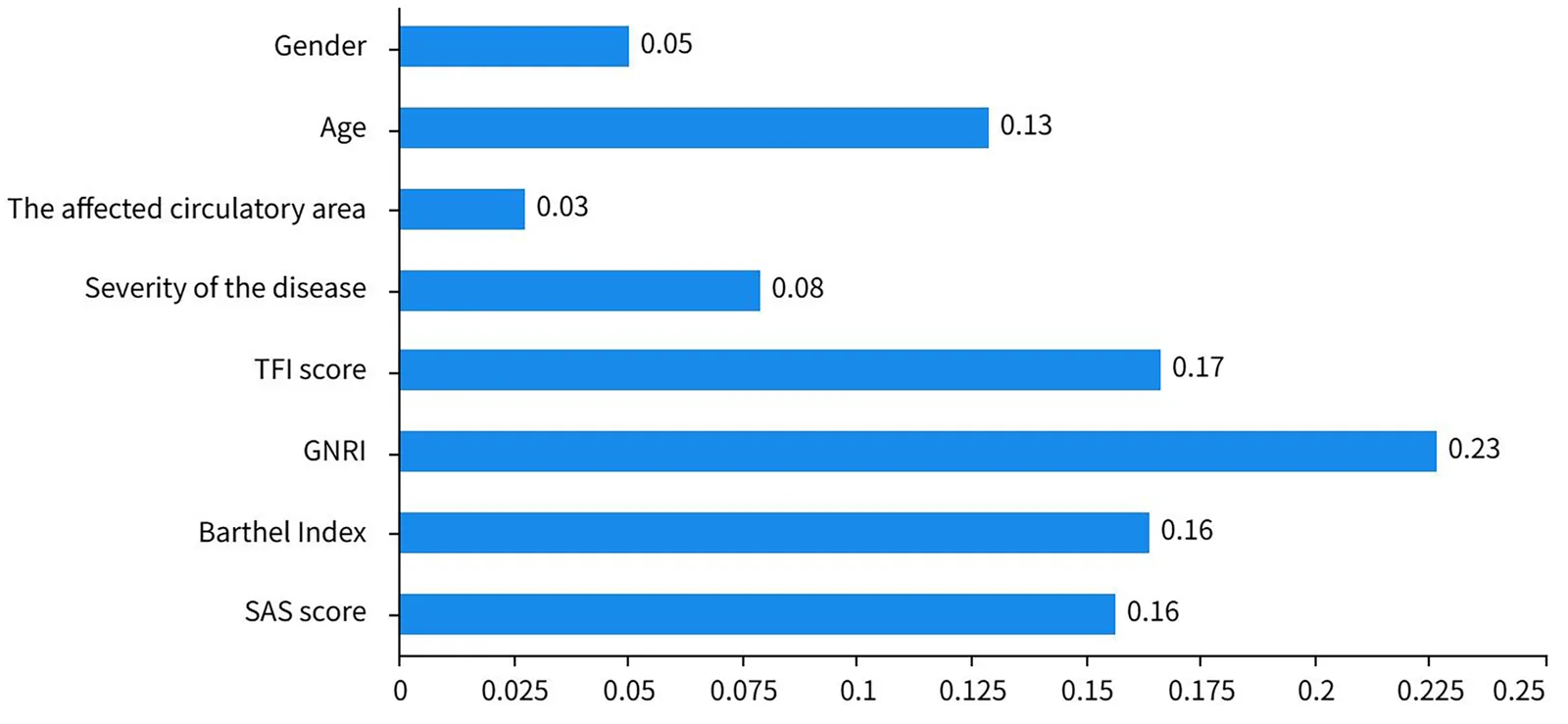
*XGBoost* figure.

**Figure 3:**
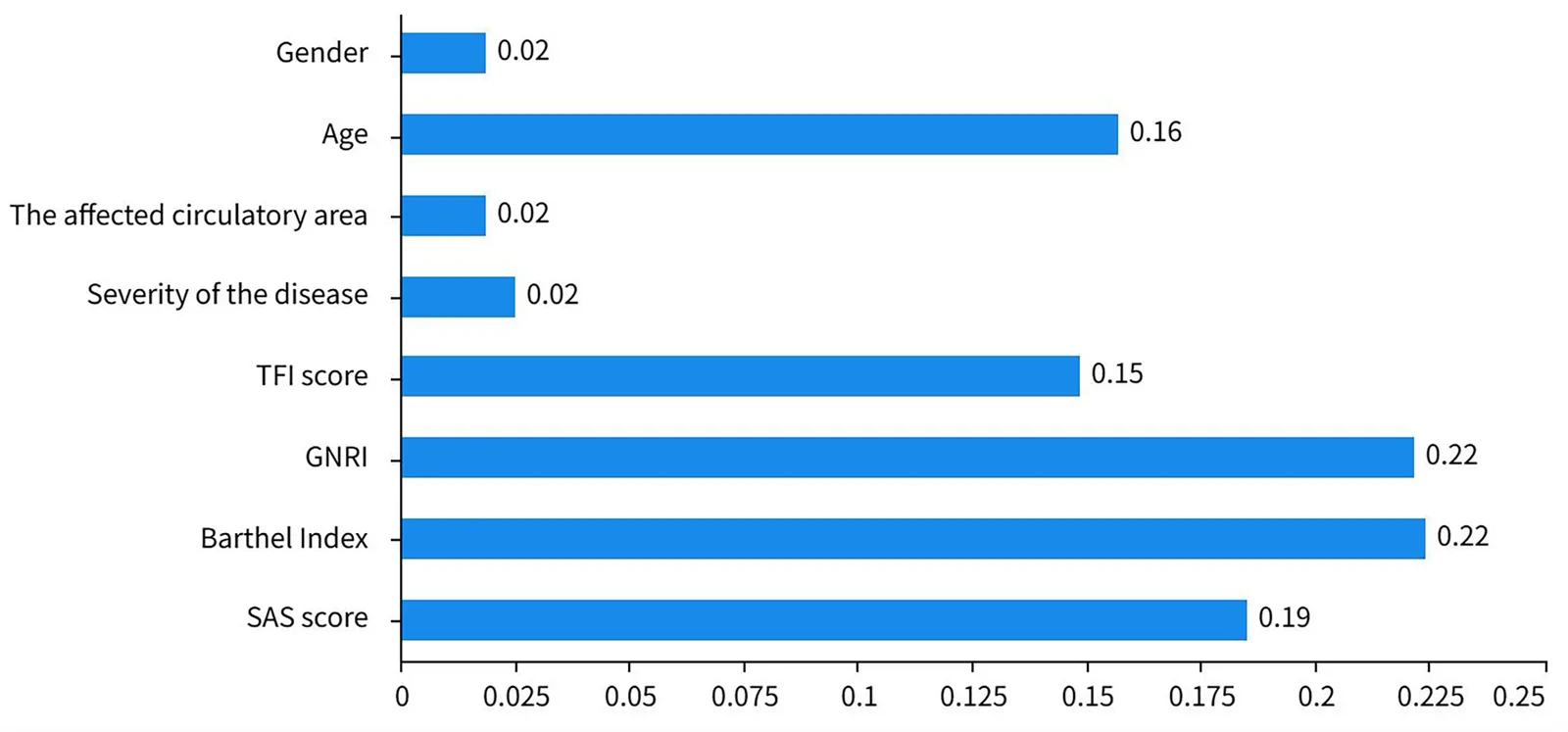
*RF* model.

### 3.3 Logistic regression analysis of intercept variables for osteoporosis in frail elderly patients with cerebral infarction

To further validate the reliability of risk factors for osteoporosis in frail elderly patients with cerebral infarction, variables selected by LASSO, XGBoost, and RF machine learning were incorporated into logistic regression analysis. Results indicated that age, TFI score, and SAS score were all risk factors for osteoporosis in this patient group (OR = 1.102, 95% CI: 1.018–1.193; OR = 2.876, 95% CI: 1.665–4.968; OR = 1.177, 95% CI: 1.069–1.295, respectively; all P < 0.05), while GNRI and Barthel Index were protective factors (OR = 0.913, 95% CI: 0.851–0.980; OR = 0.838, 95% CI: 0.747–0.940, respectively; both P < 0.05). The Hosmer-Lemeshow goodness-of-fit test confirmed the original hypothesis that the model fits the observed data well, χ² = 2.345, P = 0.969 > 0.05. The original hypothesis was accepted, indicating that the model passed the H-L test with good goodness-of-fit, as shown in **Table 2**.

**Table 2:** Logistic regression analysis of intersection variables for osteoporosis in elderly patients with frailty complicated with cerebral infarction.

Index	Regression coefficient	Standard error	Waldχ2	*P* value	OR value	95%CI
Age	0.097	0.040	5.772	0.016	1.102	1.018~1.193
TFI score	1.057	0.279	14.356	<0.001	2.876	1.665~4.968
GNRI	-0.091	0.036	6.415	0.011	0.913	0.851~0.980
Barthel Index	-0.177	0.059	9.115	0.003	0.838	0.747~0.940
SAS score	0.163	0.049	11.118	0.001	1.177	1.069~1.295

### 3.4 Development of a risk prediction regression model for osteoporosis in elderly patients with frailty and cerebral infarction

Based on the aforementioned statistical analysis results, the following regression model was established:In(p/(1-p)) = -12.266 + 0.097*Age Time + 1.057*TFI Score - 0.091* GNRI - 0.177*Barthel Index + 0.163*SAS Score. Using the five independent predictors identified through screening, a visual scoring system was developed. Each risk factor corresponds to an independent scale axis, with the scale length intuitively reflecting the factor’s contribution weight to prognosis. The total score is calculated by summing the values corresponding to each variable. This total score is then mapped onto a probability scale to yield an individualized risk prediction value. The AUC was 0.955 (95% CI: 0.920–0.991), enabling quantitative assessment of osteoporosis risk in elderly frail patients with cerebral infarction. See **Figure 4**.

**Figure 4:**
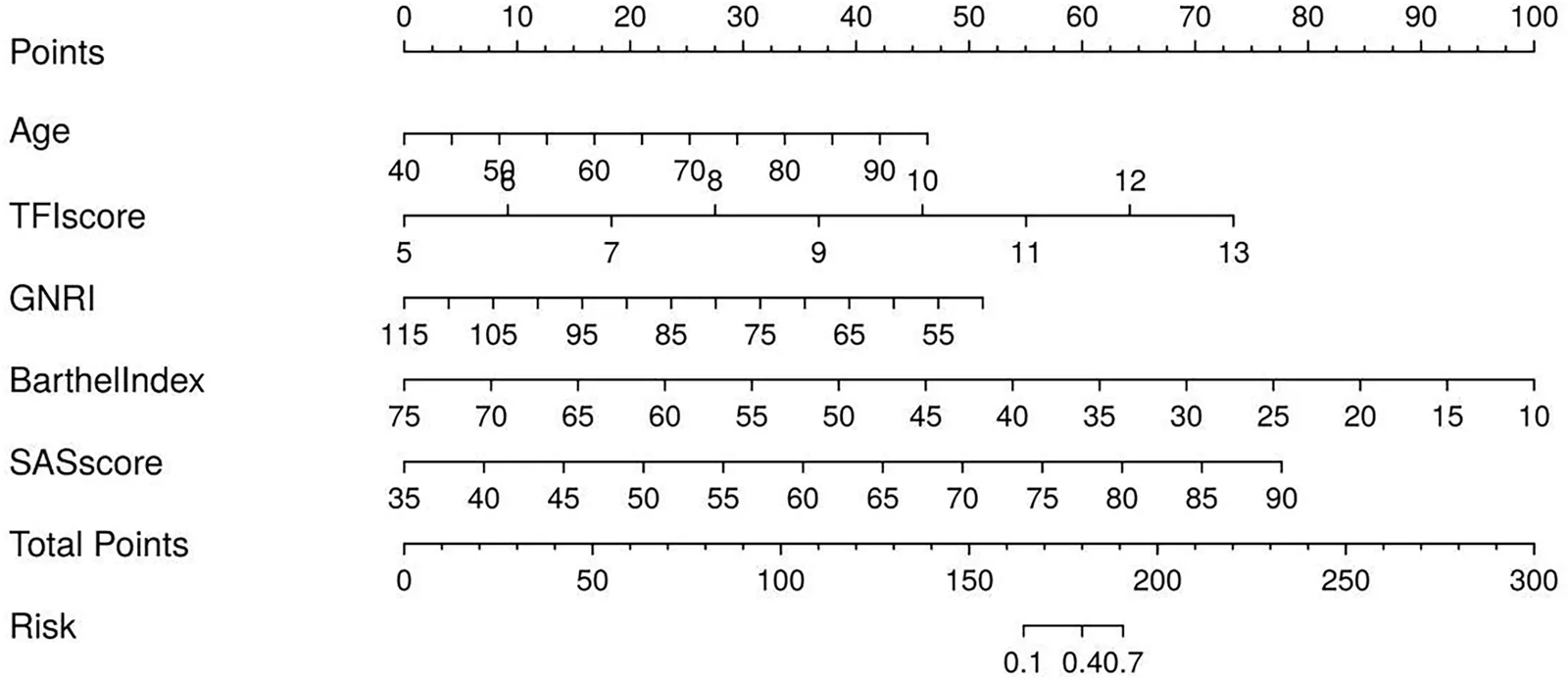
A nomogram for risk prediction of osteoporosis in elderly patients with frailty complicated with cerebral infarction. The variable labels in the figure are presented as “TFiscore” and “BarthelIndex”, which correspond to the Tilburg Frailty Inventory (TFI) score and the Barthel Index, respectively.

### 3.5 Model performance evaluation

The model was validated through 1000 repeated Bootstrap samples. The calibration curve showed a Cox-Snell R² of 0.755 and a Nagelkerke R² of 0.546, indicating strong explanatory power for the dependent variable and good calibration (see **Figure 5**). The calibration curve visually demonstrated good agreement between predicted probabilities and observed outcomes, which we considered the primary evidence for acceptable model calibration. The Hosmer-Lemeshow test yielded χ²=2.345, P = 0.969, also supporting adequate fit; however, we acknowledge that this test has limited power in small samples and should be interpreted with caution. The clinical decision curve generally exceeded both extreme curves, demonstrating superior performance compared to the extreme curves. Nagelkerke R²: 0.546, indicating strong explanatory power for the dependent variable and good calibration (**Figure 5**). The clinical decision curve generally lies above both extreme curves, demonstrating that the factors included in the nomogram provide a high net benefit for predicting osteoporosis in patients (**Figure 6**).

**Figure 5:**
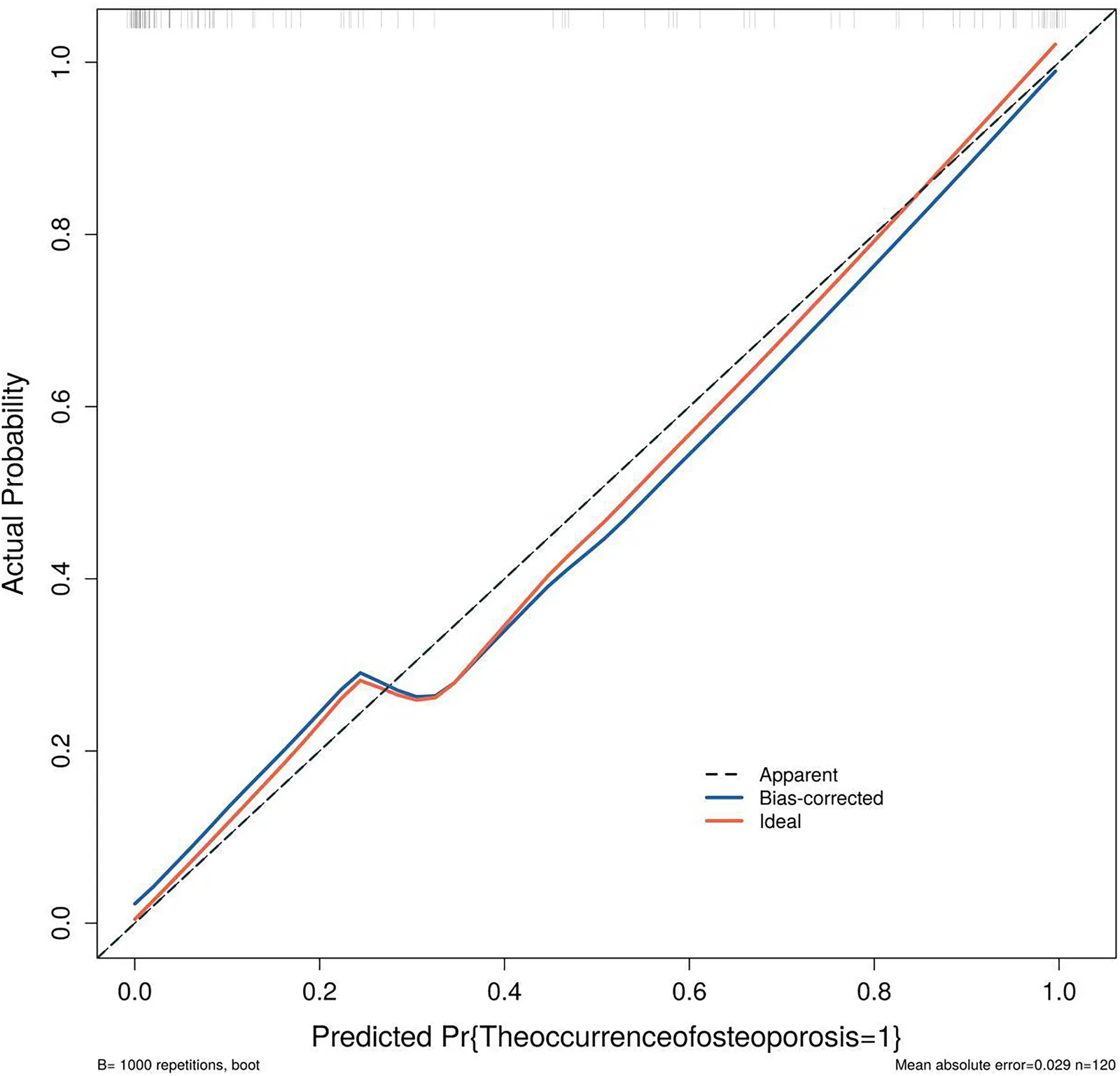
Calibration curve of the nomogram model.

**Figure 6:**
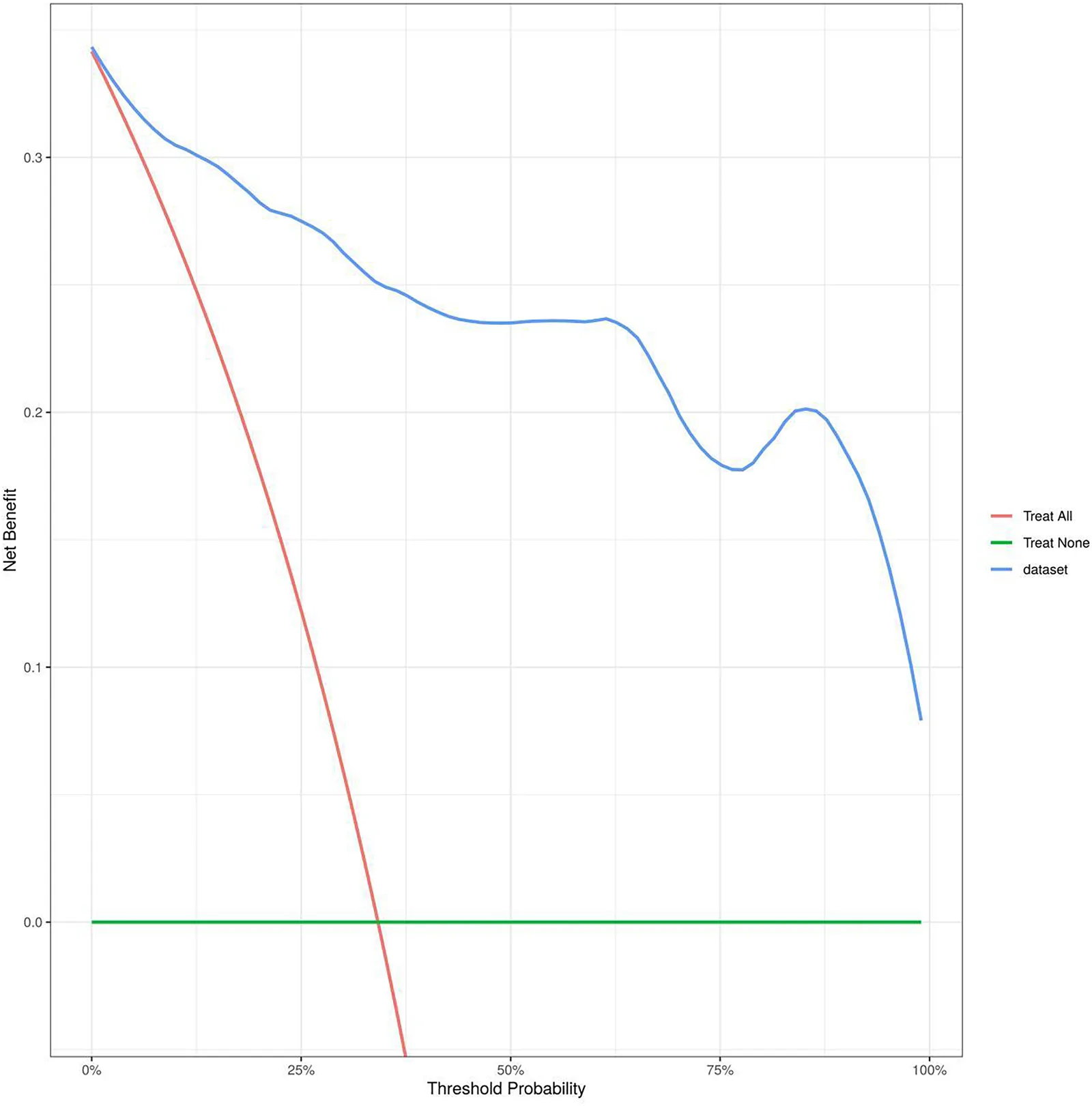
The decision curve of the nomogram model.

## 4 Discussion

As the aging of society continues to deepen, the incidence of age-related chronic diseases such as osteoporosis, diabetes, and cardiovascular diseases has been increasing annually. Extensive clinical and basic experimental research has confirmed that cerebral infarction patients experience interrupted cerebral blood supply, neuronal damage, impaired neural conduction pathways, disrupted brain functional reorganization, altered brain structure, damage to sensory neural pathways or related cortical structures, and impaired cerebral blood circulation. These factors collectively elevate the risk of concomitant osteoporosis (22, 23). Liu J et al. (24) indicated that osteoporosis in frail elderly patients with cerebral infarction can influence bone tissue changes, reduce bone mineral content, and exacerbate neurological deficits, adversely affecting patients’ quality of life and clinical outcomes. Therefore, actively analyzing key factors influencing osteoporosis in frail elderly patients with cerebral infarction is crucial for implementing corresponding prevention and treatment measures to improve prognosis.

Data from this study revealed statistically significant differences (P < 0.05) between the osteoporosis group and the non-osteoporosis group in terms of gender, age, affected circulatory system region, and disease severity. This disparity is likely attributed to the progressive aging of physiological functions with increasing patient age. Examples include accelerated brain protein atrophy, bone calcium loss, and diminished limb strength, collectively leading to reduced skeletal muscle mass. This leads to weakened anabolic metabolism, increased catabolic processes, and diminished muscle regeneration capacity, triggering “metabolic resistance” (25). Furthermore, as indicated by Curtis JR et al. (26), advancing age is accompanied by a gradual decline in bone density. Oxidative stress can enhance osteoclast activity, increase bone resorption, accelerate bone loss, and elevate the risk of osteoporosis. Previous studies indicate that in women, estrogen deficiency directly leads to uninhibited osteoclast activity, reduced osteoblast function, and impaired vitamin D synthesis, accelerating rapid bone loss. Combined with women’s inherently lower muscle mass and peak bone mass compared to men, this increases muscle atrophy, stimulates skeletal mechanics, exacerbates bone loss, and heightens osteoporosis risk. Previous studies indicate that elderly patients with severe frailty and cerebral infarction require prolonged bed rest, reducing opportunities for outdoor sun exposure. This can lead to insufficient vitamin D synthesis in the body, impairing calcium absorption and increasing the risk of osteoporosis (27). Unilateral multiple infarcts in the anterior circulation indicate direct damage to the motor cortex or corticospinal tract, increasing the risk of hemiplegia or activity limitations in the affected limb. This prolongs prolonged bed rest and reduces daily activities, The lack of mechanical stress on bones activates osteoclasts and inhibits osteoblast function, triggering rapid bone loss and disuse osteoporosis, consistent with the findings of Frenkel-Toledo S et al. (28). However, data from this study indicate that gender, affected circulatory system region, and disease severity—excluded via LASSO regression, XGBoost, and RF machine learning algorithms—demonstrate limited independent predictive value for this study’s outcomes. This may stem from the indirect nature of their influence on osteoporosis, potentially mediated through intermediate variables like nutritional status or activities of daily living. Their independent contribution diminishes when more direct clinical indicators are incorporated. Furthermore, strong multicollinearity or interactions may exist among these variables and with other predictors, rendering their net effects insignificant in multivariate models. Forcibly including them may reduce model generalization. While machine algorithms supporting model parsimony and stability by excluding these variables are promising, current clinical evidence remains insufficient. Future research is needed to validate this approach.

Data from this study indicate that the TFI and SAS scores were higher in the incident group than in the non-incident group. Logistic regression analysis revealed that both indicators are risk factors for osteoporosis in elderly patients with frailty and cerebral infarction (P < 0.05). This is because the TFI score comprehensively assesses an individual’s degree of frailty by quantifying health variables such as medical history, functional status, and activities of daily living. It is crucial to recognize that frailty is clinically and pathophysiologically intertwined with sarcopenia, which is characterized by the progressive loss of skeletal muscle mass and function. Sarcopenia contributes to osteoporosis through multiple pathways: reduced mechanical loading on bones diminishes adaptive bone remodeling; decreased muscle-derived factors (myokines) that regulate bone metabolism; and shared inflammatory and endocrine derangements that promote both muscle catabolism and bone resorption. Given this complex interplay, machine learning has been increasingly applied to assess and manage risks for these geriatric syndromes, offering a promising approach for integrated risk prediction (29). Patients with high scores are in a state of severe frailty, often accompanied by multiple chronic diseases, functional decline, and limitations in activities of daily living. For example, in diabetic patients, insufficient insulin secretion or insulin resistance may lead to reduced bone formation and increased bone resorption. accumulation of advanced glycation end-products can impair collagen cross-linking, thereby reducing bone mineral density. Chronic hypoxia associated with chronic obstructive pulmonary disease may influence bone metabolism via the HIF-1α pathway. These chronic conditions may increase the risk of osteoporosis by affecting bone mineral density (30). Furthermore, patients in severe frailty often have limited mobility, making it difficult to perform effective bed-to-floor activity training. This hinders improvements in lumbar and back muscle function and bone density. Insufficient mechanical loading reduces prostaglandin E2 release in bone tissue, diminishing adaptive bone remodeling and thereby triggering osteoporosis. Previous studies have identified a positive correlation between anxiety and depression and bone density. As anxiety and depression worsen, they can cause deterioration in patients’ physical and motor functions and decline in activities of daily living, potentially leading to clinical somatic diseases. Reduced indoor and outdoor activity becomes a risk factor for bone loss and osteoporosis. Furthermore, Araújo AP et al. (31) suggest that one potential mechanism by which emotional factors reduce bone mineral content involves elevated cortisol levels. High endogenous cortisol can induce secondary osteoporosis, characterized pathologically by reduced bone formation. The underlying mechanism involves anxiety-induced increases in corticotropin-releasing hormone (CRH) and dysfunction of the pituitary-adrenal axis. where elevated glucocorticoids act on skeletal glucocorticoid receptors, increasing bone resorption and enhancing osteoclastic activity. Concurrently, heightened CRH secretion and hypercortisolemia suppress the reproductive axis and reduce gonadotropin levels, further elevating the risk of osteoporosis.

Research data indicates that the GNRI and Barthel Index scores were lower in the incident group compared to the non-incident group. Logistic regression analysis revealed that these indicators serve as protective factors against osteoporosis in elderly patients with frailty and cerebral infarction (P < 0.05). Previous studies indicate that activities of daily living (ADL) serve as a crucial indicator for assessing elderly health status. In frail elderly patients with cerebral infarction experiencing impaired ADL, reduced mobility accelerates muscle aging, ultimately leading to physical functional impairment (32). A low Barthel Index indicates impaired daily activity capacity and severe neurological deficits in frail elderly patients with cerebral infarction. Under conditions of reduced muscle mass, diminished muscle function, or weakened muscle strength, impaired nerve conduction rapidly exacerbates these conditions in the short term. Severe physical inactivity leads to decreased skeletal muscle mass, reduced muscle strength, and type II muscle fiber atrophy, thereby increasing the risk of osteoporosis. Liang F et al. (33) noted that GNRI is a relatively simple, accurate, and objective tool for assessing “nutrition-related risk” by integrating indicators such as albumin, height, and weight. Low GNRI expression indicates deficiencies in key nutrients like protein and vitamin D. Inadequate protein intake reduces bone matrix synthesis, severely impairs calcium absorption and bone mineralization, stimulates osteoclast activity, accelerates bone resorption, leads to bone mass loss, and increases osteoporosis risk. Traditional nomogram models are typically constructed solely based on logistic regression, overlooking multicollinearity and feature selection among different variables. This approach has limitations in scientific rigor and precision (34). Machine learning algorithms, as an emerging AI technology, are applied clinically due to their capability to process complex high-dimensional data. Among these, LASSO regression primarily identifies representative variables by introducing penalty terms; Random Forest (RF) enhances model generalization through out-of-bag data; XGBoost identifies key variables using second-order derivative information and penalty terms. While individual algorithms exhibit inherent biases, this study combines all three. By screening for overlapping variables and incorporating them into logistic regression, the approach synergistically enhances the model’s overall robustness, predictive power, and interpretability (35, 36). Following this logical regression integration, a nomogram prediction model is constructed. This transforms complex multi-factor regression equations into graphical representations, rendering abstract data outcomes visualizable and readable. It enables assessment of the approximate probability of osteoporosis occurrence based on various predictive variables. The calibration curve demonstrates good consistency between the nomogram’s predicted probabilities and actual occurrence rates. The decision curve indicates high clinical utility, suggesting the model provides a more reliable quantitative tool for clinical decision-making.

## 5 Conclusions

In summary, age, TFI score, GNRI, Barthel Index, and SAS score significantly influence the development of osteoporosis in elderly patients with frailty and cerebral infarction. A predictive model constructed based on these factors demonstrates high predictive value, excellent calibration, and clinical utility. Clinicians should implement scientifically effective prevention and treatment measures targeting these risk factors to improve patient outcomes. Despite these clinical contributions, this study has limitations. First, the relatively small sample size (n=120) and the single-center design resulted in a highly homogeneous sample, which may increase the risk of model overfitting and limit the generalizability of findings. Notably, with 41 outcome events and 5 final predictor variables in the logistic regression model, the events-per-variable (EPV) ratio was approximately 8.2, falling below the widely recommended threshold of 10–15 events per variable for binary logistic regression. This EPV limitation may lead to coefficient instability and overfitting, particularly in machine learning frameworks such as Random Forest and XGBoost, which generally require substantially larger sample sizes to generate reliable feature importance metrics. To mitigate these concerns, we implemented rigorous hyperparameter tuning with grid search and 10-fold cross-validation, and used the ‘overlap-coverage’ screening strategy to select only the most robust variables. Furthermore, the observed AUC of 0.955, while encouraging, should be interpreted with caution. This high discrimination value likely reflects some degree of optimistic bias due to the limited sample size and the inherent tendency of machine learning models to overfit in small datasets, even with our mitigation strategies. Nevertheless, the EPV limitation warrants caution when interpreting the model’s effect estimates. Regarding calibration assessment, we acknowledge that relying solely on the Hosmer-Lemeshow test (P = 0.969) is insufficient to confirm good calibration, as this test has limited statistical power in small samples and may fail to detect true miscalibration. We therefore placed greater emphasis on the calibration curve, which visually demonstrated good agreement between predicted and observed probabilities (Cox-Snell R²=0.755, Nagelkerke R²=0.546), supporting acceptable model calibration. Second, and most critically, the predictive model’s performance was assessed solely through internal bootstrap validation. We emphasize that bootstrap validation, while valuable for internal assessment, cannot replace independent external validation. The optimistic performance metrics, including the high AUC, may not be reproducible in external populations without further validation. The absence of an external validation cohort means its true generalizability and clinical utility remain unconfirmed. As emphasized in recent high-quality studies, external validation is an indispensable step for translating machine learning-based prediction models into clinical practice (9). Future research should optimize study design with larger, multi-center cohorts and incorporate external validation to enhance the reliability and scientific rigor of conclusions.

## Data Availability

The original contributions presented in the study are included in the article/supplementary material. Further inquiries can be directed to the corresponding authors.
